# E2 Site Mutations in S Protein Strongly Affect Hepatitis B Surface Antigen Detection in the Occult Hepatitis B Virus

**DOI:** 10.3389/fmicb.2021.664833

**Published:** 2021-11-10

**Authors:** Hao Wang, Fenfang Liao, Junmo Xie, Wenbo Gao, Min Wang, Jieting Huang, Ru Xu, Qiao Liao, Zhengang Shan, Yourong Zheng, Xia Rong, Chengyao Li, Yongshui Fu

**Affiliations:** ^1^Guangzhou Blood Center, Guangzhou, China; ^2^Department of Transfusion Medicine, School of Laboratory Medicine and Biotechnology, Southern Medical University, Guangzhou, China

**Keywords:** hepatitis B virus, occult hepatitis B infection, mutation, hepatitis B surface antigen, HBsAg secretion impairment

## Abstract

The mechanism of occult hepatitis B infection (OBI) has not yet been fully clarified. Our previous research found that novel OBI-related mutation within S protein, E2G, could cause the hepatitis B surface antigen (HBsAg) secretion impairment, which resulted in intracellular accumulation in OBI of genotype B. Here, to further explore the role of E2 site mutations in the occurrence of OBI, we analyzed these site mutations among 119 OBI strains identified from blood donors. Meanwhile, 109 wild-type HBV strains (HBsAg positive/HBV DNA positive) were used as control group. Furthermore, to verify the E2 site mutations, two conservative 1.3-fold full-gene expression vectors of HBV genotype B and C (pHBV1.3B and pHBV1.3C) were constructed. Then, the E2 mutant plasmids on the basis of pHBV1.3B or pHBV1.3C were constructed and transfected into HepG2 cells, respectively. The extracellular and intracellular HBsAg were analyzed by electrochemical luminescence and cellular immunohistochemistry. The structural characteristics of S proteins with or without E2 mutations were analyzed using relevant bioinformatics software. E2 mutations (E2G/A/V/D) existed in 21.8% (26/119) of OBIs, while no E2 mutations were found in the control group. E2G/A/V/D mutations could strongly affect extracellular and intracellular level of HBsAg (*p* < 0.05). Notably, unlike E2G in genotype B that could cause HBsAg intracellular accumulation and secretion decrease (*p* < 0.05), E2G in genotype C could lead to a very significant HBsAg decrease both extracellularly (0.46% vs. pHBV1.3C) and intracellularly (11.2% vs. pHBV1.3C) (*p* < 0.05). Meanwhile, for E2G/A mutations, the relative intracellular HBsAg (110.7–338.3% vs. extracellular) and its fluorescence intensity (1.5–2.4-fold vs. with genotype-matched pHBV1.3B/C) were significantly higher (*p* < 0.05). Furthermore, N-terminal signal peptides, with a typical cleavage site for peptidase at positions 27 and 28, were exclusively detected in S proteins with secretion-defective mutants (E2G/A). Our findings suggest that: (1) E2G/A/V/D mutations were confirmed to significantly influence the detection of HBsAg, (2) the underlying mechanism of OBI caused by E2G mutation is quite different between genotype B and genotype C, and (3) E2G/A could produce a N-terminal truncated S protein, which might attribute to the HBsAg secretion impairment in the OBIs.

## Introduction

Hepatitis B virus (HBV) infection is a global health problem. Despite the availability of HBV vaccine, approximately 2 billion people have been infected with HBV (Lozano et al., 2012), and 350–500 million people are chronically infected worldwide (Lamontagne et al., 2016). Researchers have identified at least eight HBV genotypes (A–H), among which genotypes B and C are the most prevalent in China (Zeng et al., 2005; Li et al., 2013). HBV has a small (3.2 kb), partially double-stranded, mutation-prone DNA genome with four overlapping open reading frames, pre-S1/S2/S, pre-C-C, P, and X. The pre-S1/S2/S encodes the three hepatitis B surface antigen (HBsAg) proteins, termed large, middle, and small surface antigen protein (Lamontagne et al., 2016). In particular, the small surface antigen (S protein) is the predominant HBsAg protein, which consists of 226 amino acids (aa) and encoded by S gene (nt155–832). HBsAg is an important serological biomarker for the detection of HBV, which is present on the surface of 22 nm subviral particles (SVPs) and 42 nm virions. The SVPs appear as spheres or filaments composed of empty envelopes and are secreted 10^3^-fold to 10^6^-fold excess over virions (Hu and Liu, 2017), hence HBsAg is virtually equivalent to SVPs. SVPs are synthesized at the rough endoplasmic reticulum (ER) and secreted via the constitutive secretory pathway of the host cell (Watanabe et al., 2007; Jiang et al., 2015).

Occult HBV infection (OBI), as a special form of HBV infection, is characterized by the persistence of HBsAg seronegativity and positive of HBV DNA (usually less than 200 IU/ml) (Raimondo et al., 2008), and it is potentially associated with increased incidence of acute exacerbation, cirrhosis, and hepatocellular carcinoma (Raimondo et al., 2010; Said, 2011; Kwak and Kim, 2014; Makvandi, 2016). The mechanism resulting in OBI is complicated that remains to be clarified (Samal et al., 2012). Mutations in HBV S gene is one of the factors that contribute to the occurrence of OBI (Samal et al., 2012). Recently, our research has found some novel OBI-related mutations within S gene, among which E2G was the most noteworthy mutation because it led to the intracellular accumulation and secretion decrease of HBsAg in OBI of genotype B (Wang H. et al., 2020). In the present study, to further explore the role of E2 mutations in the occurrence of OBI, we looked for all the E2 mutations in OBI of genotypes B and C. In addition, plasmids containing these E2 mutations were employed to investigate the role of these mutations accounted for the detection or secretion of HBsAg and HBV virions *in vitro*.

## Materials and Methods

### Specimen Collection, DNA Extraction, PCR, and Sequencing

Specimen collection, DNA extraction, PCR, and sequencing were carried out as previously described (Wang H. et al., 2020). Briefly, 119 OBI plasma samples, including 89 with genotype B and 30 with genotype C, were collected from voluntary blood donors at Guangzhou Blood Center during April 2015–May 2017. The OBI blood donors were diagnosed according to the standard described previously (Allain et al., 2010; Zheng et al., 2011; Wang H. et al., 2020). The control group consisted of 109 HBsAg (+)/HBV DNA (+) blood donors (57 with genotype B and 52 with genotype C) randomly collected from the Guangzhou Blood Center. The HBV genotypes were determined by phylogenetic analysis as previously reported (Tang et al., 2018). Plasma samples of all subjects were stored at −40°C until used. All participants in this study have provided their informed consent. This study has been approved by the Medical Ethics Committee of Guangzhou Blood Center. HBV DNA was harvested from 2.5 ml of plasma using the High Pure Viral Nucleic Acid Extraction kit (Roche Diagnostic, Germany) and amplified with nested PCRs targeting for the Pre-S/S region or for the full-length genome as described previously (Candotti et al., 2008; Zahn et al., 2008; Tang et al., 2018). PCR amplicons were sequenced by Invitrogen (Guangzhou, China). The nucleotide sequences were aligned using BioEdit 7.0 software, and deduced amino acid sequences of the S protein were compared with the genotype-specific consensus sequence of the control group.

### Construction of Conservative Plasmid of Hepatitis B Virus Genotype B and Genotype C

The plasmid of HBV genotype B (pHBV1.3B) was constructed as described previously (Zhang et al., 2015), and have been fully used and verified in our previous studies (Wang H. et al., 2020), while the plasmid of HBV genotype C (pHBV1.3C) was constructed following the methods and principles of pHBV1.3B, and have been fully verified capable of genome replication and expression of viral proteins. Briefly, two 1.3-fold consensus sequences (nucleotides 1038–3215/1–1984) of HBV genotypes B and C were chemically synthesized (Sangon Biotech Shanghai, China), and cloned into *Hin*dIII/*Eco*RI digested pcDNA3.1(+) vector referred to as pHBV1.3B and pHBV1.3C, respectively.

### Site-Specific Mutagenesis

To obtain the E2 single-point-mutant plasmids (pHBV1.3B/C-E2N) based on pHBV1.3B or pHBV1.3C, a Site-directed Gene Mutagenesis Kit (Beyotime Biotechnology, China) was used according to the manufacturer’s instruction. The corresponding specific primers used for site-specific mutagenesis are listed in Supplementary Table 1. The pHBV1.3B/C or pHBV1.3B/C-E2N was extracted by the plasmid midi kit (TaKaRa, China) and confirmed by sequencing.

### Cell Cultures and Transfection

HepG2 cells were maintained as described previously (Wang H. et al., 2020) and seeded at 1.0 × 10^5^ cells in 12-well plates. Twenty-four hours later, E2 mutant plasmids or pHBV1.3B/C were transfected into HepG2 cells (1 μg/well) using Neofect DNA Transfection Reagent (Neofect Biotech, China). For the detection of extracellular and intracellular HBsAg, the lysates of the cells and culture supernatant were harvested at 48 h post-transfection. For quantitation of supernatant HBV DNA, to exclude the influence of plasmid on the HBV DNA detection, the culture supernatant was removed at 4 h post-transfection and the cells were washed five times with 1×PBS, then adding another 1 ml of fresh medium to the cells, which were collected 48 h post-transfection. Three independent transfection experiments were performed in duplicate for each E2 mutant plasmid.

### Quantification of Extracellular and Intracellular Hepatitis B Surface Antigen

At 48 h post-transfection, the culture supernatants were collected for detecting the expression level of HBsAg secreted by the HepG2 cells. The transfected cells were washed three times with 1×PBS before lysing with cell lysis buffer (Beyotime Biotechnology, China). The level of HBsAg production in culture supernatants and cell lysates were tested by electrochemiluminescence immunoassay (ECIA) using the fully automated Elecsys HBsAg II assay (Roche, Mannheim, Germany) (Gencay et al., 2018) according to the manufacturer’s recommendations.

### Detection of Supernatant Hepatitis B Virus DNA

Culture supernatant was collected 48 h post-transfection and incubated at 37°C for 15 min with 1 U/ml of DNase I (Promega, United States) to remove free DNA. Then, the supernatant HBV DNA were quantitated using a commercial HBV DNA real-time quantitative PCR kit (Daan Gene, China) with a lower detection limit of 200 IU/ml according to the manufacturer’s instructions. The qPCR reactions were performed on a Real-Time PCR System (Applied Biosystems ABI 7500, United States). The cycling conditions were as follows: 93°C 2 min, 10 cycles of 93°C for 45 s and 55°C for 60 s, followed by 30 cycles of 93°C for 30 s and 55°C for 45 s.

### Immunofluorescence Staining

Immunofluorescence (IF) staining and fluorescence intensity analysis of intracellular HBsAg were performed as described previously (Wang H. et al., 2020). In brief, HepG2 cells were grown on glass coverslips and transfected with E2 mutant plasmids or pHBV1.3B/C. Transfected cells were washed with 1×PBS prior to fixation with 4% paraformaldehyde. The fixed cells were permeabilized with 0.25% Triton X-100 and blocked with 1% bovine serum albumin. Then cells were incubated with diluted (1:500) rabbit anti-HBsAg antibody (Abcam, England) at 4°C overnight. Then the cells were washed three times with 1×PBS before incubation with CY3-conjugated (red) secondary antibodies (goat anti-rabbit) (Affinity Biosciences, China) at room temperature for 1 h. Cell nuclei were stained with DAPI. The staining cells were analyzed by Leica microscope (DMI3000) at a magnification of ×200 or ×400. Fluorescence intensity was analyzed using ImageJ software (NIH, Bethesda, MD, United States). All images used for fluorescence intensity were captured with the same imaging parameters.

### Bioinformatics Analysis

The structural characteristics (signal peptides, physicochemical properties, transmembrane domains, and secondary structures) of S proteins with or without E2 mutations were analyzed as previously reported (Wang et al., 2019; Santos Junior et al., 2020). Protein signal peptide prediction was carried out on the SignalP v5.0 server^1^. The physicochemical properties were obtained by ExPASy^2^. The presence of transmembrane regions was verified by the TMHMM v2.0^3^. Secondary structure analysis was performed by SOPMA^4^.

### Statistical Analysis

Fisher’s exact test was used to compare the differences of all the E2 mutations in OBI and control group. One-way ANOVA, followed by Fisher’s LSD test, was used for the comparison of the differences between E2 mutant plasmids and pHBV1.3B/C on extracellular and intracellular HBsAg. The quantitative results of HBV DNA level were log transformed and further analyzed by Mann–Whitney *U* test. Statistical analysis was carried out using GraphPad Prism 7.0 (GraphPad Software, United States). *P*-values less than 0.05 (two-tailed) were considered statistically significant.

## Results

### E2 Mutations in the S Gene

Mutation E2G/A/V/D were identified in 1.1–11.2% of the genotype B OBIs, and E2G/A/D were identified in 3.3–13.3% of the genotype C OBIs (Table 1). Overall, E2 mutations (E2G/A/V/D) existed in 21.8% (26/119) of the OBI subjects, while no E2 mutation was found in the control group. Among these E2 mutations, E2G was significantly frequent in the OBIs compared with the control group (*p* < 0.05), while no significant difference was found regarding other types of E2 mutation between the two groups (*p* > 0.05) (Table 1).

**TABLE 1:** E2 mutations in OBI and control group.

**Mutation**	**No. (%)**		***p***	**No. (%)**		***p***
	**OBI B *n* = 89**	**HBV** **B *n* = 57**		**OBI C *n* = 30**	**HBV C *n* = 52**	
E2G	10 (11.2)	0	0.0191*	4 (13.3)	0	0.0186*
E2A	7 (7.8)	0	0.0597	1 (3.3)	0	0.3417
E2V	2 (2.2)	0	0.4362	–	–	–
E2D	1 (1.1)	0	0.6587	1 (3.3)	0	0.3417
Total	20 (22.4)	0	0.0005	6 (20)	0	0.0033

The S protein amino acid sequences of OBI and control group were compared with the genotype-matched consensus sequence of the control group. E2 mutations in OBI and control group were examined using Fisher’s exact test. *p < 0.05.

### Extracellular and Intracellular Hepatitis B Surface Antigen

**FIGURE 1:**
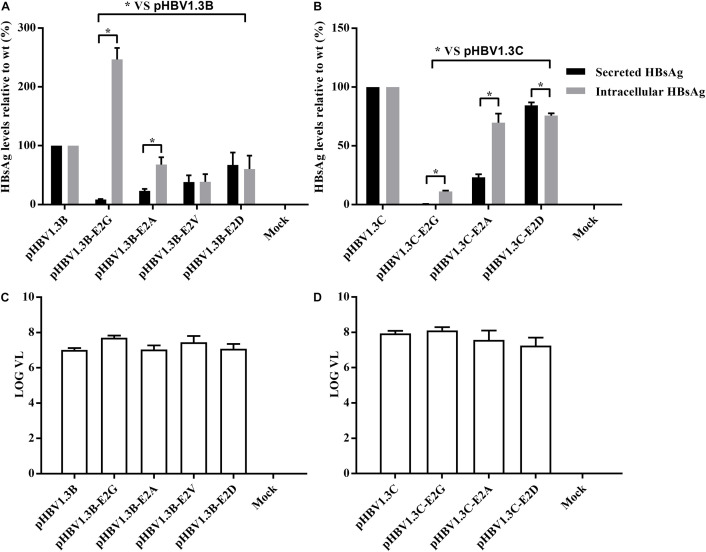
Analysis of HBsAg and HBV DNA production by electrochemiluminescence and real-time PCR. **(A,B)** Extracellular and intracellular HBsAg production in HepG2 cells transfected with E2 mutant plasmids (pHBV1.3B/C-E2N) and pHBV1.3B/C. The relative expression of extracellular and intracellular HBsAg in pHBV1.3B/C-E2N was compared with that of genotype-matched pHBV1.3B/C. Data are demonstrated as means ± SD from three independent experiments. *p < 0.05. Mock: pcDNA3.1(+) vector. **(C,D)** Quantitation of supernatant HBV DNA with real-time quantitative PCR. pHBV1.3B/C-E2N and pHBV1.3B/C were transfected into HepG2 cells, and supernatants were analyzed using real-time quantitative PCR at 48 h after transfection. The quantitative results of HBV DNA levels were log transformed. Data were demonstrated as median and interquartile range from three independent experiments. Mock: pcDNA3.1(+) vector.

**FIGURE 2:**
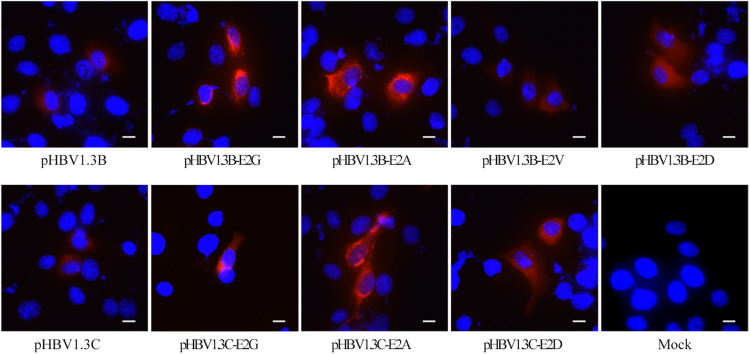
Immunofluorescence staining of HBsAg in HepG2 cells transfected with pHBV1.3B/C and E2 mutated plasmid. The staining of HepG2 cells transfected with pHBV1.3B/C showed a diffuse fine particle staining, while cells transfected with pHBV1.3B/C-E2G/A gathered in the perinuclear region or showed a flaked and coarse grains staining pattern. The staining of cells transfected with pHBV1.3B-E2V/D and pHBV1.3C-E2D was similar to that of genotype-matched pHBV1.3B/C. The cells were fixed at 48 h after transfection and staining with a monoclonal rabbit antibody to the HBsAg, followed by an anti-rabbit IgG conjugated with CY3 (red). The cell nuclei were stained with DAPI (blue). The scale bar stands for 10 μm. Magnification, ×200 or ×400. Mock: pcDNA3.1(+) vector.

To further confirm the role of E2 mutations in OBIs, we transfected HepG2 cells with pHBV1.3B/C-E2N, pHBV1.3B, and pHBV1.3C. The extracellular and intracellular HBsAg were then analyzed. The results showed that all of the E2 mutations could strongly affect extracellular and intracellular HBsAg level compared with pHBV1.3B/C (*p* < 0.05). The relative HBsAg level in supernatant of E2 mutations significantly decreased by 15.5–91.5% (*p* < 0.05). The intracellular relative HBsAg level of pHBV1.3B-E2G significantly increased by 146.7%, while other E2 mutations significantly decreased by 24.3–88.8% (*p* < 0.05) (Figures 1A,B and Supplementary Table 2). Notably, unlike pHBV1.3B-E2G, which could cause HBsAg intracellular accumulation (246.7% vs. pHBV1.3B) and HBsAg secretion decrease (8.5% vs. pHBV1.3B) (*p* < 0.05), pHBV1.3C-E2G could lead to a very significant HBsAg decrease both extracellularly (0.5% vs. pHBV1.3C) and intracellularly (11.2% vs. pHBV1.3C) (*p* < 0.05). Meanwhile, the intracellular relative level of HBsAg transfected with pHBV1.3B/C-E2G/A was significantly increased than extracellular (110.7–338.3% vs. extracellular), while pHBV1.3C-E2D was significantly decreased than extracellular (91.2% vs. extracellular) (*p* < 0.05) (Figures 1A,B and Supplementary Table 2). Hence, pHBV1.3B/C-E2G/A could decrease HBsAg secretion, whereas pHBV1.3C-E2D promoted HBsAg secretion. Immunofluorescence results showed that HBsAg were expressed in cells transfected with E2-mutant plasmids and pHBV1.3B/C (Figure 2).

### Supernatant Hepatitis B Virus DNA

The supernatant HBV DNA of HepG2 cells transfected with pHBV1.3B-E2N (median range, 6.9–7.7 log10 IU/ml) and pHBV1.3C-E2N (median range, 7.0–8.1 log10 IU/ml) did not have significant differences with genotype-matched pHBV1.3B (median, 7.0 log10 IU/ml) or pHBV1.3C (median, 7.9 log10 IU/ml) (*p* > 0.05) (Figures 1C,D). The results showed that E2 mutations did not significantly affect the secretion of HBV virions.

### Immunofluorescence of E2 Mutated Hepatitis B Surface Antigen

The results shown in Figures 1A,B revealed that pHBV1.3B/C-E2G/A may lead to HBsAg-blocking compared with the pHBV1.3B/C. To further explore this issue, we analyzed the subcellular distribution and fluorescence density of E2 mutated HBsAg by immunofluorescence.

As shown in Figure 2, pHBV1.3B/C-transfected HepG2 cells showed a diffuse fine particle staining, while the pHBV1.3B/C-E2G/A-transfected cells gathered in the perinuclear region or showed a flaked and coarse grains staining pattern. The staining of pHBV1.3B-E2V/D or pHBV1.3C-E2D-transfected cells was similar to that of genotype-matched pHBV1.3B/C.

**FIGURE 3:**
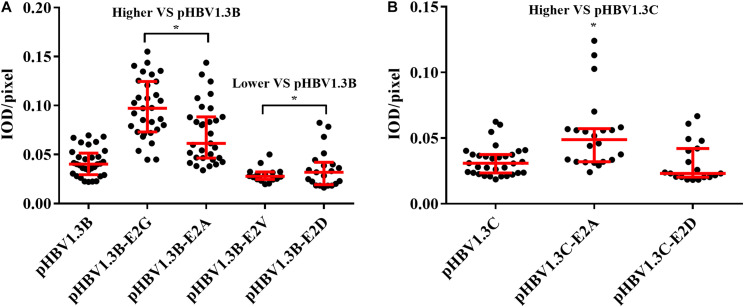
Comparison of fluorescence intensity of HBsAg between E2 mutated plasmid and pHBV1.3B/C. Compared with pHBV1.3B, the fluorescence density of cells transfected with pHBV1.3B-E2G and pHBV1.3B-E2A was significantly higher (*p* < 0.0001), while pHBV1.3B-E2V and pHBV1.3B-E2D was significantly lower (*p* < 0.05) **(A)**. Compared with pHBV1.3C, the fluorescence density of cells transfected with pHBV1.3C-E2A was significantly higher (*p* < 0.001). No significant changing was found between pHBV1.3C-E2D and pHBV1.3C **(B)**. The positive fluorescent cells transfected with pHBV1.3C-E2G were too few to be included in the fluorescence intensity statistical analysis. Fluorescence density = integrated optical density (IOD)/pixel. Integrating optical density (IOD) refers to the sum of the optical density values of each pixel in the area of the cross-sectional image of the cell. *p < 0.05. Mock: pcDNA3.1(+) vector.

Immunofluorescence density analysis showed that, compared with pHBV1.3B (median, 0.040 IOD/pixel), the pHBV1.3B-E2G-transfected cells (median, 0.097 IOD/pixel) and pHBV1.3B-E2A-transfected cells (median, 0.061 IOD/pixel) were significantly higher (1.5–2.4-fold vs. pHBV1.3B) (*p* < 0.0001), while pHBV1.3B-E2V (median, 0.027 IOD/pixel) and pHBV1.3B-E2D (median, 0.031 IOD/pixel) were significantly lower (*p* < 0.05) (Figure 3A). Compared with pHBV1.3C (median, 0.030 IOD/pixel), the fluorescence density of pHBV1.3C-E2A-transfected cells (median, 0.048 IOD/pixel) was significantly higher (1.6-fold vs. pHBV1.3C) (*p* < 0.001). However, no significant changing was found in pHBV1.3C-E2D (median, 0.023 IOD/pixel) (Figure 3B). The positive fluorescent cells transfected with pHBV1.3C-E2G were too few to involve in the fluorescence density analysis.

### Bioinformatics Analysis

The signal peptide, physicochemical properties, transmembrane structure, and secondary structure of the S proteins with or without E2 mutations were analyzed using bioinformatics tools. The results showed that there are typical N-terminal signal peptides in S proteins with E2G/A mutations in genotypes B and C, with a typical cleavage site (CS) for peptidase at positions 27 and 28 (Supplementary Figure 2). No significant difference was found in S proteins with E2 mutations during physicochemical property, transmembrane domain, and secondary structure analysis.

## Discussion

The pathogenesis of OBI is multi-factorial and has not been fully understood. This study investigated a series of S protein E2 mutations in OBI genotypes B and C, which play an important role in the development of OBI.

In the present study, beside the S protein mutation E2G in OBI genotype B we previously reported (Wang H. et al., 2020), a significantly more frequent E2G mutation was also identified in 13.3% of OBI genotype C strains (*p* < 0.05). Meanwhile, other kinds of E2 mutations (E2A/V/D) were observed in 1.1–7.8% of OBI strains (*p* > 0.05) (Table 1). In general, E2 mutations are specific and representative OBI-related mutations were found in 21.8% (*p* < 0.05) of OBI subjects and none in the control group (Table 1), which is consistent with a previous study in China (Wang J. et al., 2020). Functional analysis showed that nearly all of the E2 mutations could significantly decrease extracellular and intracellular HBsAg level (except E2G in genotype B with increased intracellular HBsAg level) (*p* < 0.05) (Figures 1A,B), which could partly explain the lack of HBsAg detection that characterized the occult HBV carriage *in vivo*. Previously, the mutations associated with OBI have often been identified in HBV S region (Khan et al., 2004; Ito et al., 2010; Kalinina et al., 2010; Yuan et al., 2010; Candotti et al., 2012; Huang et al., 2012; Martin et al., 2012), especially in the major hydrophilic region (El Chaar et al., 2010; Huang et al., 2012), while mutation at N terminus of the S protein is so rarely reported that only a few studies have found E2 mutations in OBI (Wang J. et al., 2020).

On the other hand, the relative intracellular HBsAg of E2G/A were significantly increased than extracellular (110.7–338.3% vs. extracellular) (*p* < 0.05) (Figure 1 and Supplementary Table 2). Meanwhile, the fluorescence density of pHBV1.3B-E2G/A and pHBV1.3C-E2A was significantly higher than that of genotype-matched pHBV1.3B/C (Figure 3) (*p* < 0.0001), and the staining pattern of E2G/A mutated HBsAg (Figure 2) is consistent with that of HBsAg intracellular accumulation reported previously (Pong Kian et al., 2005; Wang H. et al., 2020). All these results suggested that E2G/A induce the HBsAg intracellular accumulation, which might be related to its secretion impairment. Impaired HBsAg secretion is involved in the occurrence of OBI (Biswas et al., 2013). Previous researches have documented some OBI-related mutations that could affect the secretion of HBsAg (Ito et al., 2010; Martin et al., 2012; Wu et al., 2012). Of note is that the significantly lower fluorescence density in pHBV1.3B-E2V/D-transfected cells (Figure 3A) might relate to the decrease of intracellular HBsAg without relative intracellular accumulation (Figure 1A and Supplementary Table 2). In addition, pHBV1.3C-E2D could promote HBsAg secretion relatively (Figure 1B), whereas the fluorescence density of its transfected cells (median, 0.023 IOD/pixel) was not significantly lower (*p* > 0.05) (Figure 3B), which may be due to its weak effect on promoting HBsAg secretion.

Interestingly, a considerable difference was found in the effect of E2G on the expression of intracellular and extracellular HBsAg between genotypes B and C (Figures 1A,B). Our previous reports (Wang H. et al., 2020) and the present analyses (Figures 1A,B, 2, 3) demonstrated that E2G in OBI genotype B could cause HBsAg intracellular accumulation and HBsAg secretion decrease (*p* < 0.05). However, in genotype C, E2G could lead to a very significant HBsAg decrease both extracellularly (0.5% vs. pHBV1.3C) and intracellularly (11.2% vs. pHBV1.3C) (*p* < 0.05) (Figures 1A,B). These results suggest that there are great differences in the underlying mechanism of OBI caused by E2G between genotype B and genotype C. In this study, as the E2 mutant plasmids were constructed based on pHBV1.3B and pHBV1.3C, there are 7.6% (244 nt/3215 nt) differences between these two whole genomes (data not shown). Some gene structural differences may be involved in this interesting distinction, which needs to be further investigated.

In addition to the undetectable HBsAg, extremely low viral load is another common feature of OBI (Raimondo et al., 2008). However, the OBI-specific E2 mutations did not affect the secretion of HBV virions compared with pHBV1.3B/C (*p* > 0.05) (Figures 1C,D). Actually, HBV virions and subviral particles (the main components of HBsAg) are produced and released in different pathways during HBV replication cycle (Hu and Liu, 2017; Seitz et al., 2020), and not all the OBI-related mutations affect HBV DNA expression *in vitro* (Garcia et al., 2009; Wu et al., 2010; Huang et al., 2012; Chen et al., 2016). Therefore, E2 mutations studied here strongly influenced the detection of HBsAg, but not virions.

Noteworthy, although E2 mutations did not significantly affect HBV virions, most of the OBI individuals with E2 mutations have low HBV DNA levels (data not shown). On the other hand, HBsAg could be detected in the culture supernatants transfected with E2 mutant plasmids (Figures 1, 2), which is not consistent with the characteristics of negative HBsAg in OBI blood donors. Actually, we observed that most OBI sequences (with or without E2 mutations) have multiple mutations in S region (Wang H. et al., 2020). In this context, the combination of those mutations may play a coordinated role in determining the presentation of OBI (Svicher et al., 2012; Biswas et al., 2013; Chen et al., 2016; Wang H. et al., 2020), which needs to be further investigated.

Another interesting novel finding from the present study was that N-terminal signal peptides were exclusively detected in S proteins with secretion-defective mutants mentioned previously (E2G/A) (Supplementary Figure 2). As we all know, signal peptide is a short peptide attached to the N-terminal of the protein cleaved off by signal peptidase during translation. Under normal conditions, the 226 aa S protein precursor was shown to transmembrane and translocated at the ER compartment by its first transmembrane domain (TM-I) located between residues 8 and 22 of N-terminal (Suffner et al., 2018), which is not cleaved by any of the signal peptidases intracellularly (Patient et al., 2010; Suffner et al., 2018). Then the S protein precursors are assembled into mature spherical particles and transported by the constitutive secretory pathway (Bukau et al., 2006; Watanabe et al., 2007; Jiang et al., 2015). However, in this study, S proteins with secretion-defective mutations (E2G/A) lead to a signal peptide cleavage between amino acid positions 27 and 28 (Supplementary Figure 2). The N-terminal truncated S protein may potentially affect the S protein transmembrane in ER, and thereby failing to meet the productive folding requirement of mature S protein. Unfolded or misfolded S proteins will be excluded from secretion and accumulate in ER (Hurtley and Helenius, 1989; Ma and Hendershot, 2003; Rutkowski and Kaufman, 2004; Malhotra and Kaufman, 2007). Consistent with this hypothesis, S protein with deletions in the TM-I has been reported to impair the HBsAg secretion (Prange et al., 1992).

There are several novelties of this research compared with other similar studies. First, the E2 mutants assessed in this study are rarely reported and had not been included in previous functional analysis of HBsAg. Moreover, to minimize the influence of random mutations in wild-type HBV plasmid vector, two representative 1.3-fold full-length HBV vectors (pHBV1.3B/C) based on the consensus sequences of China were used as backbones of E2 mutant expression vectors. On the other hand, however, there were also some limitations to this study. First of all, further studies will be needed to elucidate the different mechanisms of OBI genotype B and genotype C caused by E2G. Second, it remains unclear why the extent of HBsAg secretion impairment caused by E2G/A varies greatly (110.7–338.3% vs. extracellular) and why pHBV1.3C-E2D could rather promote HBsAg secretion.

## Conclusion

The current study revealed several S protein E2 mutants exist in OBI blood donors of genotypes B and C, which confirmed to be specific and representative OBI-related mutations and significantly affect the detection of HBsAg. Subsequently, there are great differences in the underlying mechanism of OBI attributed to the E2G mutations between genotype B and genotype C. Preliminary data suggested that E2G/A may produce a truncated S protein by the formation of N-terminal signal peptide, which might cause the HBsAg secretion impairment in OBI. The results obtained from this study might be informative for understanding the occurrence of OBI.

## Data Availability Statement

The original contributions presented in the study are included in the article/Supplementary Material, further inquiries can be directed to the corresponding author/s.

## Ethics Statement

The studies involving human participants were reviewed and approved by the Medical Ethics Committee of Guangzhou Blood Center. The patients/participants provided their written informed consent to participate in this study.

## Author Contributions

HW and YF conceived and designed the experiments. MW, JH, RX, QL, and ZS implemented specimen collection, DNA extraction, PCR amplification, and sequencing. HW, JX, WG, and FL conducted cell cultures and transfection, and detected the HBsAg and HBV DNA. HW and FL conducted statistical analyses and drafted the first manuscript. YZ, XR, CL, and YF gave support and supervised the research. XR, YF, and CL helped to draft the manuscript and revised it. All authors contributed to the manuscript and approved the submitted version.

## Conflict of Interest

The authors declare that the research was conducted in the absence of any commercial or financial relationships that could be construed as a potential conflict of interest.

## Publisher’s Note

All claims expressed in this article are solely those of the authors and do not necessarily represent those of their affiliated organizations, or those of the publisher, the editors and the reviewers. Any product that may be evaluated in this article, or claim that may be made by its manufacturer, is not guaranteed or endorsed by the publisher.

## Footnotes

1
http://www.cbs.dtu.dk/services/SignalP/

2
http://web.expasy.org/protparam/

3
http://www.cbs.dtu.dk/services/TMHMM/

4
https://npsa-prabi.ibcp.fr/cgi-bin/npsa_automat.pl?page=npsa_sopma.html
