# Supplementary material for: E2 Site Mutations in S Protein Strongly Affect Hepatitis B Surface Antigen Detection in the Occult Hepatitis B Virus
Source: Front Microbiol. 2021 Nov 10;12:664833. doi: 10.3389/fmicb.2021.664833 (PMC8635997; doi:10.3389/fmicb.2021.664833)
Supplement: Supplementary file 1 [file Data_Sheet_1.PDF]

(A)

```
                        10        20        30        40        50        60        70        80        90       100
                    ....|....|....|....|....|....|....|....|....|....|....|....|....|....|....|....|....|....|....|....|
pHBV1.3B            MENIASGLLGPLLVLQAGFFLLTKILTIPQSLDSWWTSLNFLGGTPVCLGQNSQSQISSHSPTCCPPICPGYRWMCLRRFIIFLCILLLCLIFLLVLLDY
pHBV1.3B-E2G        .G..................................................................................................
pHBV1.3B-E2A        .A..................................................................................................
pHBV1.3B-E2V        .V..................................................................................................
pHBV1.3B-E2D        .D..................................................................................................

                       110       120       130       140       150       160       170       180       190       200
                    ....|....|....|....|....|....|....|....|....|....|....|....|....|....|....|....|....|....|....|....|
pHBV1.3B            QGMLPVCPLIPGSSTTSTGPCKTCTTPAQGTSMFPSCCCTKPTDGNCTCIPIPSSWAFAKYLWEWASVRFSWLSLLVPFVQWFVGLSPTVWLSVIWMMWF
pHBV1.3B-E2G        ....................................................................................................
pHBV1.3B-E2A        ....................................................................................................
pHBV1.3B-E2V        ....................................................................................................
pHBV1.3B-E2D        ....................................................................................................

                       210       220
                    ....|....|....|....|....|.
pHBV1.3B            WGPSLYNILSPFMPLLPIFFCLWVYI
pHBV1.3B-E2G        ..........................
pHBV1.3B-E2A        ..........................
pHBV1.3B-E2V        ..........................
pHBV1.3B-E2D        ..........................
```

(B)

```
                        10        20        30        40        50        60        70        80        90       100
                    ....|....|....|....|....|....|....|....|....|....|....|....|....|....|....|....|....|....|....|....|
pHBV1.3C            MENTTSGFLGPLLVLQAGFFLLTRILTIPQSLDSWWTSLNFLGGAPTCPGQNSQSPTSNHSPTSCPPICPGYRWMCLRRFIIFLFILLLCLIFLLVLLDY
pHBV1.3C-E2G        .G..................................................................................................
pHBV1.3C-E2A        .A..................................................................................................
pHBV1.3C-E2D        .D..................................................................................................

                       110       120       130       140       150       160       170       180       190       200
                    ....|....|....|....|....|....|....|....|....|....|....|....|....|....|....|....|....|....|....|....|
pHBV1.3C            QGMLPVCPLLPGTSTTSTGPCKTCTIPAQGTSMFPSCCCTKPSDGNCTCIPIPSSWAFARFLWEWASVRFSWLSLLVPFVQWFVGLSPTVWLSVIWMMWY
pHBV1.3C-E2G        ....................................................................................................
pHBV1.3C-E2A        ....................................................................................................
pHBV1.3C-E2D        ....................................................................................................

                       210       220
                    ....|....|....|....|....|.
pHBV1.3C            WGPSLYNILSPFLPLLPIFFCLWVYI
pHBV1.3C-E2G        ..........................
pHBV1.3C-E2A        ..........................
pHBV1.3C-E2D        ..........................
```

Supplementary Figure 1. Sequence alignment of S protein with or without E2 mutations. Sequence alignment of S protein (226aa) with or without E2 mutations in genotype B (A) and genotype C (B). The nucleotide sequence of S region of pHBV1.3B and pHBV1.3C was translated into amino acid sequence for alignment.
