# Supplementary material for: E2 Site Mutations in S Protein Strongly Affect Hepatitis B Surface Antigen Detection in the Occult Hepatitis B Virus
Source: Front Microbiol. 2021 Nov 10;12:664833. doi: 10.3389/fmicb.2021.664833 (PMC8635997; doi:10.3389/fmicb.2021.664833)
Supplement: Supplementary file 2 [file Data_Sheet_2.PDF]

Signal peptides in S proteins with or without E2 mutations

SignalP-5.0 prediction (Eukarya): pHBV1.3B

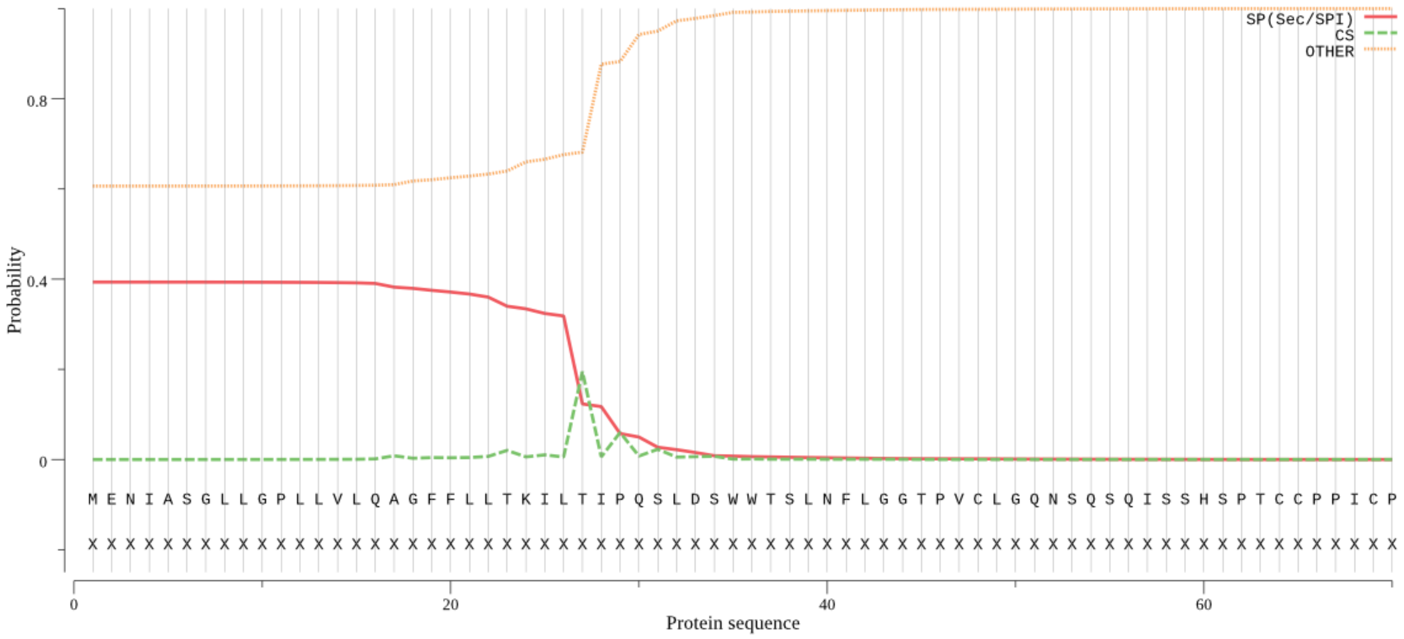

SignalP-5.0 prediction (Eukarya): pHBV1.3B-E2G

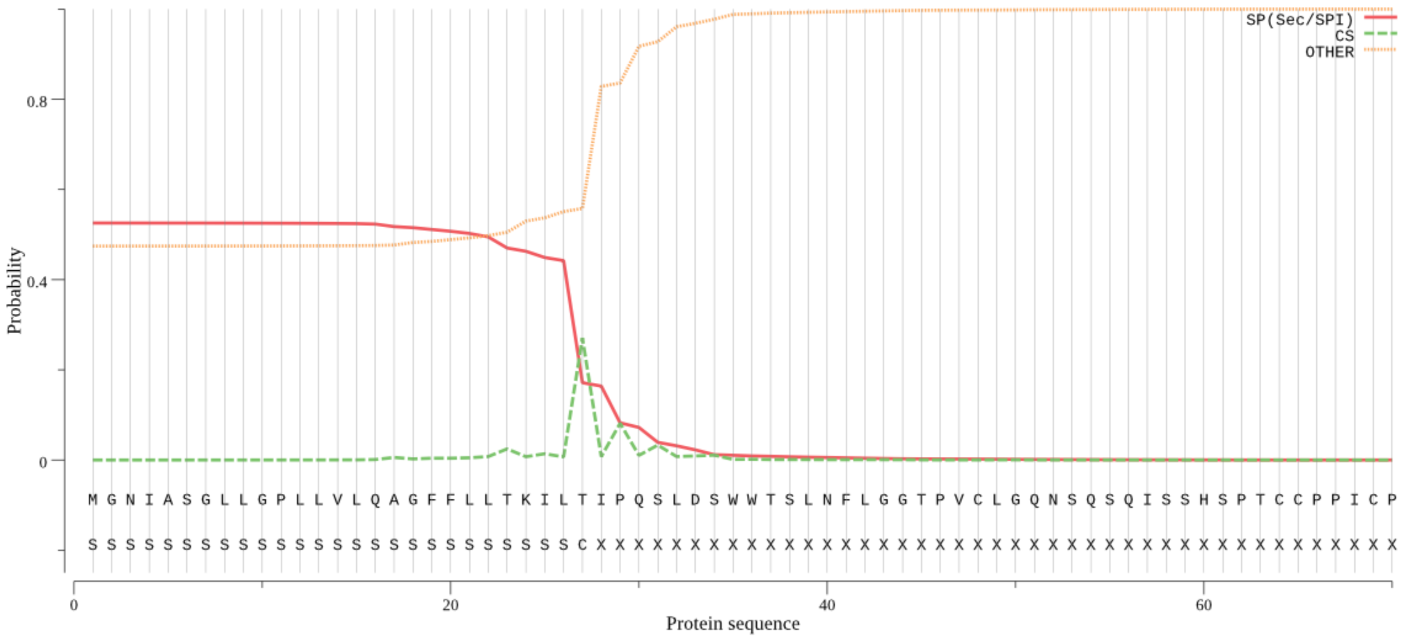

SignalP-5.0 prediction (Eukarya): pHBV1.3B-E2A

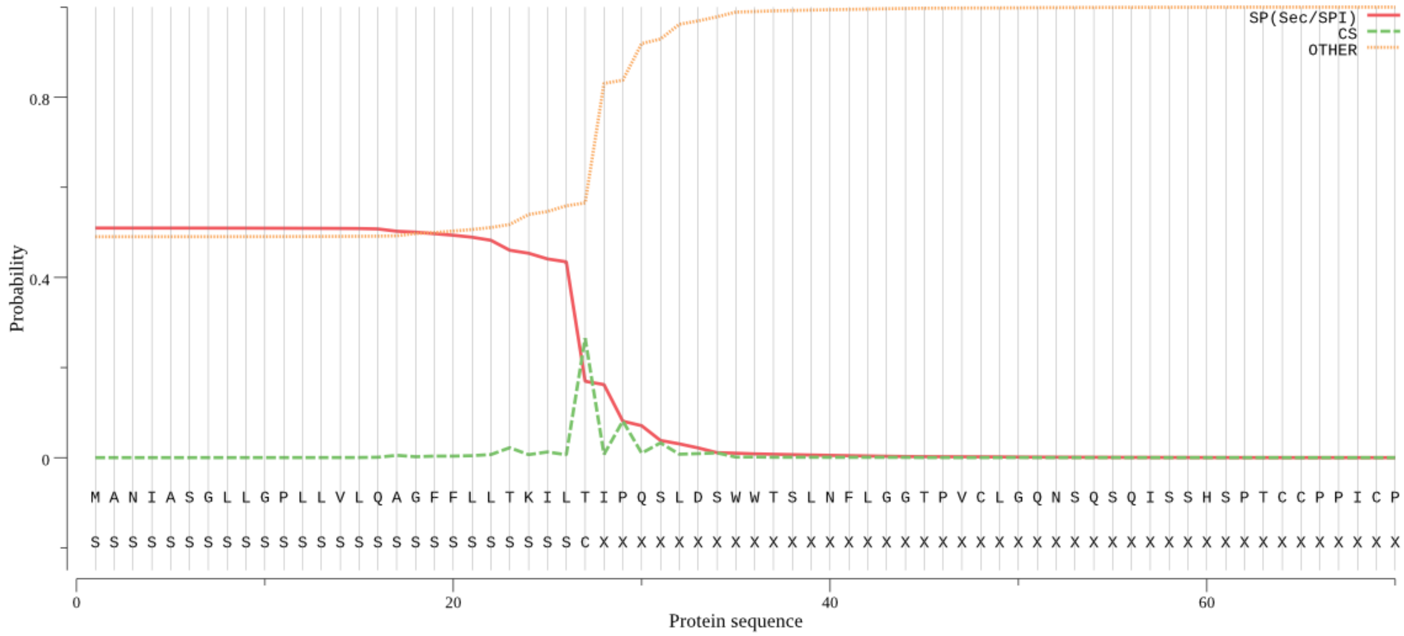

SignalP-5.0 prediction (Eukarya): pHBV1.3B-E2V

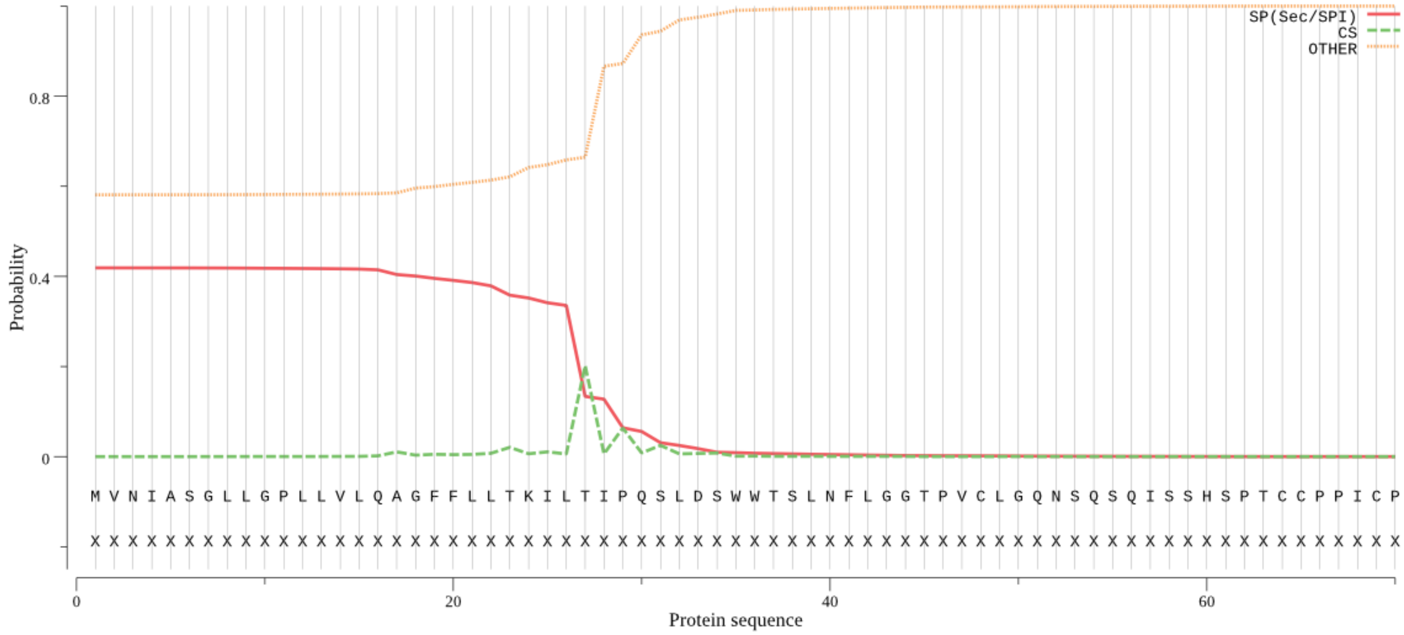

SignalP-5.0 prediction (Eukarya): pHBV1.3B-E2D

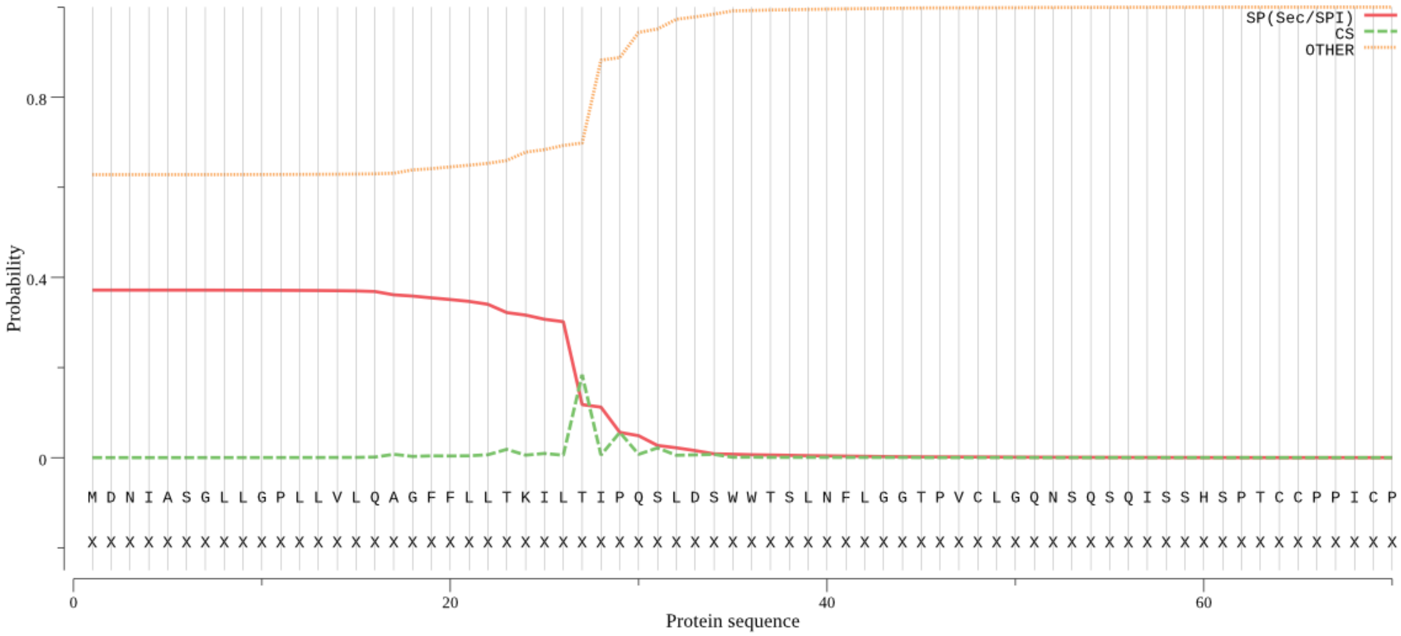

SignalP-5.0 prediction (Eukarya): pHBV1.3C

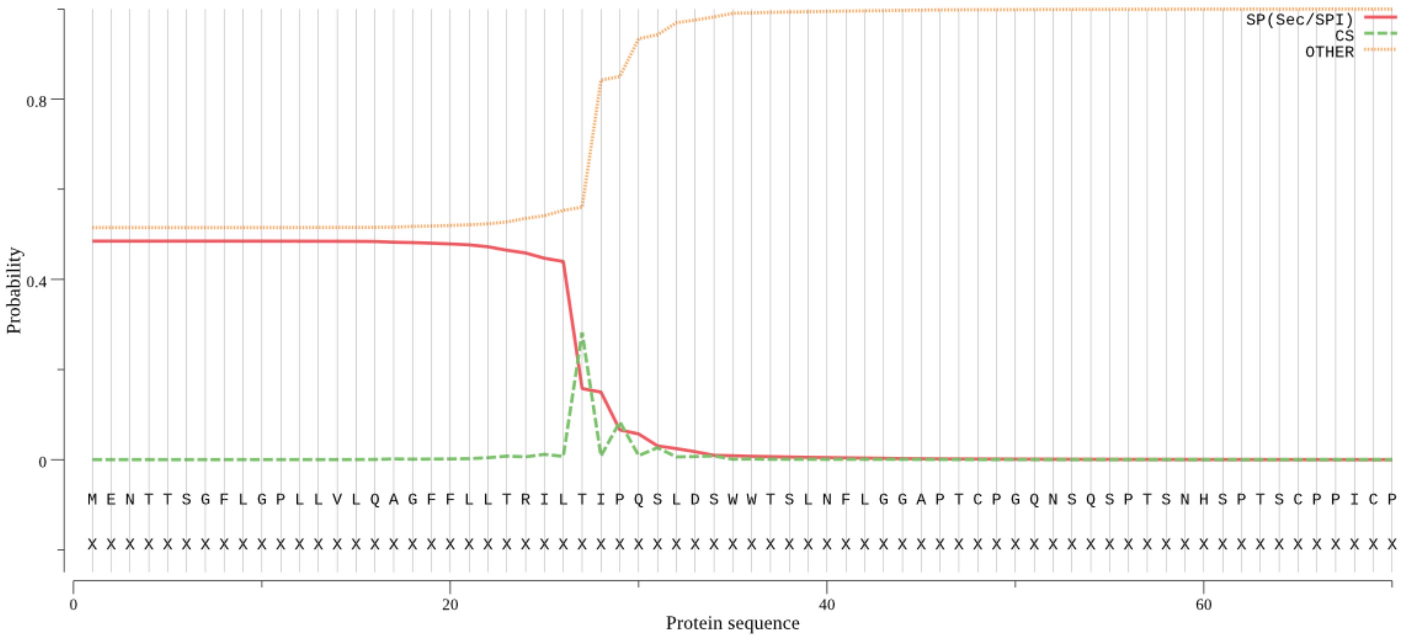

SignalP-5.0 prediction (Eukarya): pHBV1.3C-E2G

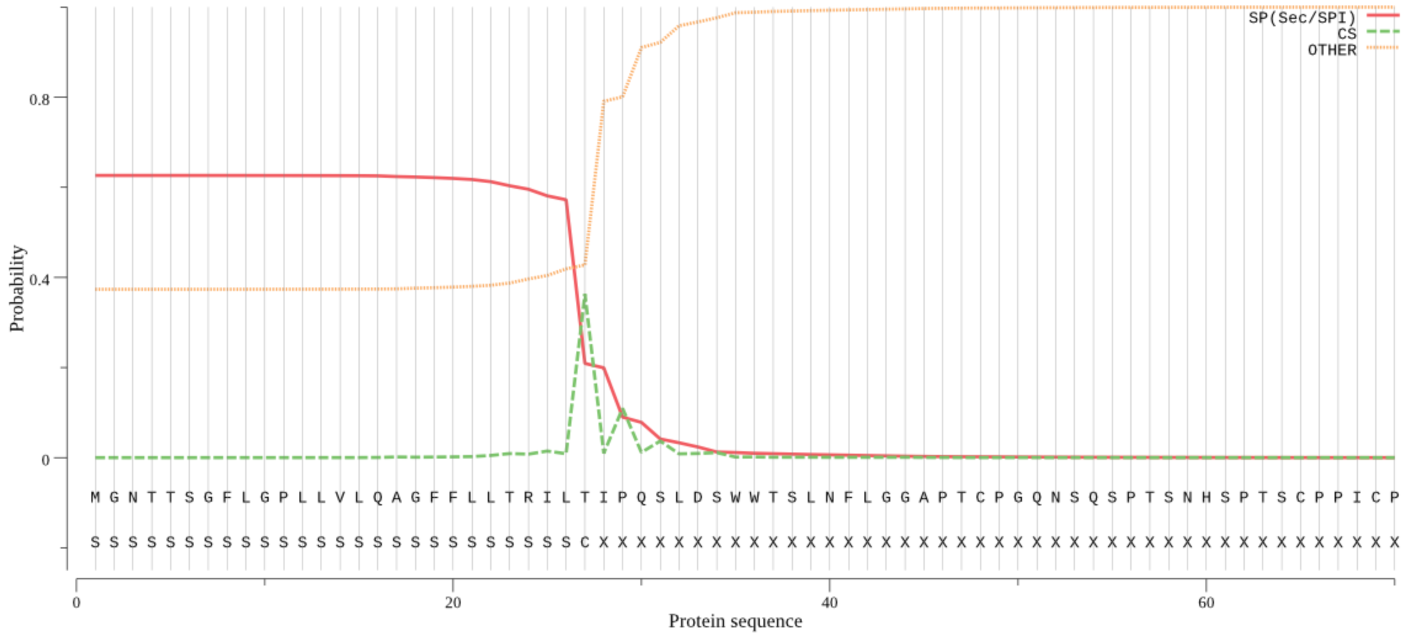

SignalP-5.0 prediction (Eukarya): pHBV1.3C-E2A

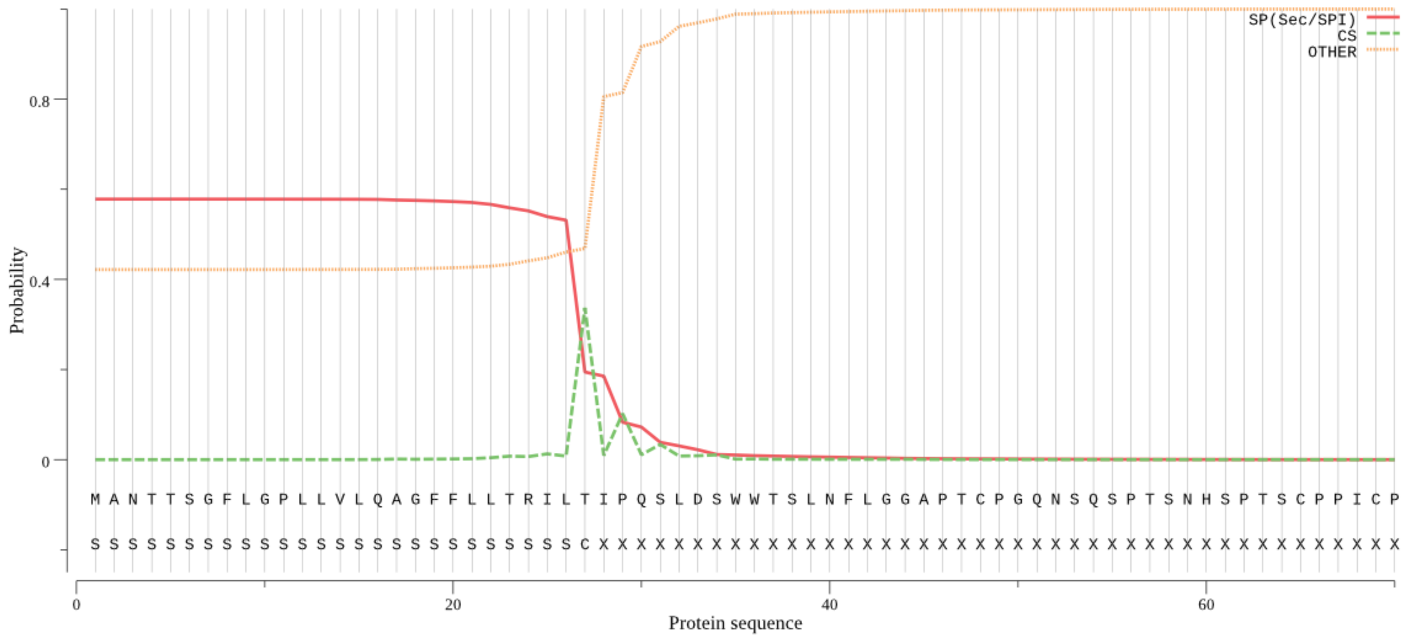

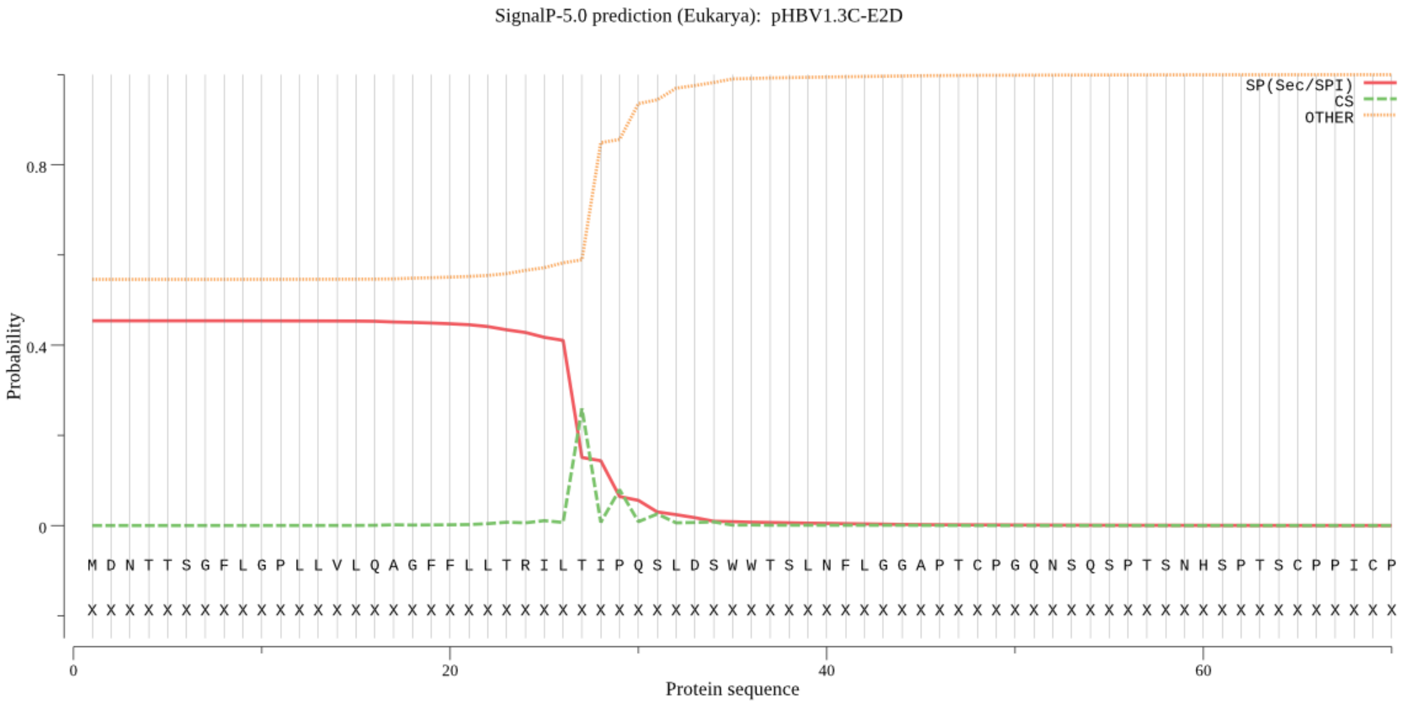

Supplementary Figure 2. SignalP were utilized in order to predict the signal peptides and location of their cleavage site in S proteins with or without E2 mutations. Predicted signal peptide (red) values equal to or greater than 0.5 (threshold) were considered of signal peptide with "S" under the corresponding amino acids. The green peak represents the typical cleavage site (CS). The results showing there are typical signal peptides in S proteins with E2G/A of genotype B and C, with typical cleavage sites for peptidase at position 27 and 28.
