# Supplementary material for: E2 Site Mutations in S Protein Strongly Affect Hepatitis B Surface Antigen Detection in the Occult Hepatitis B Virus
Source: Front Microbiol. 2021 Nov 10;12:664833. doi: 10.3389/fmicb.2021.664833 (PMC8635997; doi:10.3389/fmicb.2021.664833)
Supplement: Supplementary file 3 [file Table_1.DOCX]

| **Primers for constructing recombinant mutant vector** | | | |
| --- | --- | --- | --- |
| Genotype | Mutation | Primer | Sequence (5'–3') |
| B | E2G | Forward | AACATGG**G**GAACATCGCATCAGGAC |
|  |  | Reverse | ATGCGATGTTC**C**CCATGTTCGGTAC |
|  | E2A | Forward | AACATGG**C**GAACATCGCATCAGGAC |
|  |  | Reverse | ATGCGATGTTC**G**CCATGTTCGGTAC |
|  | E2V | Forward | AACATGG**T**GAACATCGCATCAGGAC |
|  |  | Reverse | ATGCGATGTTC**A**CCATGTTCGGTAC |
|  | E2D | Forward | AACATGGA**C**AACATCGCATCAGGAC |
|  |  | Reverse | ATGCGATGTT**G**TCCATGTTCGGTAC |
| C | E2G | Forward | AACATG**G**GGAACACAACATCAGGAT |
|  |  | Reverse | ATGTTGTGTTC**C**CCATGTTCGGTGC |
|  | E2A | Forward | AACATG**C**GGAACACAACATCAGGAT |
|  |  | Reverse | ATGTTGTGTTC**G**CCATGTTCGGTGC |
|  | E2D | Forward | AACATGA**C**GAACACAACATCAGGAT |
|  |  | Reverse | ATGTTGTGTT**G**TCCATGTTCGGTGC |

**Supplementary table 1. Primers for constructing recombinant mutant vector.** The primers used for site-speciﬁc mutagenesis were designed according to the sequence of pHBV1.3B and pHBV1.3C following the instruction of Site-directed Gene Mutagenesis Kit. Mutated codons are underlined and mutation sites are in boldface type.
