# Supplementary material for: E2 Site Mutations in S Protein Strongly Affect Hepatitis B Surface Antigen Detection in the Occult Hepatitis B Virus
Source: Front Microbiol. 2021 Nov 10;12:664833. doi: 10.3389/fmicb.2021.664833 (PMC8635997; doi:10.3389/fmicb.2021.664833)
Supplement: Supplementary file 4 [file Table_2.DOCX]

| **Extracellular and intracellular expression of HBsAg in mutant plasmids COI (%)** | | | | | | | | | | | | | | | | | |
| --- | --- | --- | --- | --- | --- | --- | --- | --- | --- | --- | --- | --- | --- | --- | --- | --- | --- |
| **Mutation** | **Secreted HBsAg (%)** | | | **Mean Diff.  VS pHBV 1.3B/C (%)** | **Significant** | ***P*** |  | **Intracellular HBsAg (%)** | | | **Mean Diff.  VS pHBV 1.3B/C (%)** | **Significant** | ***P*** |  | **Mean Diff.  Secreted VS Intracellular  (%)** | **Significant** | ***P*** |
|  |  |  |  |  |  |  |  |  |  |  |  |  |  |  |  |  |  |
|  | **1^st^** | **2^nd^** | **3^rd^** |  |  |  |  | **1^st^** | **2^nd^** | **3^rd^** |  |  |  |  |  |  |  |
| pHBV1.3B | 501.8(100) | 732.3(100) | 725.5(100) | / | / | / |  | 30.5(100) | 50.4(100) | 44.4(100) | / | / | / |  | / | / | / |
| pHBV1.3B-E2G | 47.1(9.4) | 57.3(7.8) | 58.4(8) | 91.5 | **** | <0.0001 |  | 74.3(243.6) | 115.5(229.1) | 118.8(267.4) | -146.7 | **** | <0.0001 |  | 238.3 | **** | <0.0001 |
| pHBV1.3B-E2A | 136.6(27.2) | 153.3(20.9) | 152(21) | 76.9 | **** | <0.0001 |  | 25(82.2) | 31.3(62) | 26.5(59.7) | 32.0 | *** | 0.0003 |  | 44.9 | *** | 0.0001 |
| pHBV1.3B-E2V | 248.2(49.5) | 197.9(27) | 281.9(38.9) | 61.5 | **** | <0.0001 |  | 15.8(51.8) | 13(25.7) | 17.1(38.4) | 61.3 | **** | <0.0001 |  | 0.2 | ns | 0.9841 |
| pHBV1.3B-E2D | 460.2(91.7) | 407.7(55.7) | 399.3(55) | 32.5 | *** | 0.0003 |  | 26.4(86.5) | 23.3(46.2) | 21.6(48.7) | 39.5 | **** | <0.0001 |  | -7.0 | ns | 0.4886 |
| pHBV1.3C | 334.1(100) | 321.7(100) | 334.7(100) | / | / | / |  | 17.2(100) | 16.3(100) | 17.6(100) | / | / | / |  | / | / | / |
| pHBV1.3C-E2G | 1.4(0.4) | 1.6(0.5) | 1.6(0.5) | 99.5 | **** | <0.0001 |  | 1.9(10.7) | 2(10.9) | 1.5(12) | 88.8 | **** | <0.0001 |  | 10.7 | *** | 0.0001 |
| pHBV1.3C-E2A | 68(20.4) | 81.2(25.2) | 80.6(24.1) | 76.7 | **** | <0.0001 |  | 12.8(60.8) | 12(74.4) | 13.8(73.8) | 30.3 | **** | <0.0001 |  | 46.4 | **** | <0.0001 |
| pHBV1.3C-E2D | 279.8(83.7) | 280.7(87.3) | 276.1(82.5) | 15.5 | **** | <0.0001 |  | 13.2(73.3) | 12.5(76.9) | 16.6(76.8) | 24.3 | **** | <0.0001 |  | -8.8 | *** | 0.0009 |

**Supplementary table 2. Extracellular and intracellular expression of HBsAg in mutant plasmids COI (%)** HBsAg was measured by cutoff index (COI), <1COI was unresponsive. Blank control (pcDNA3.1) HBsAg < 1COI. * represents significant difference between the two groups; “ns” represents no significant difference between the two groups. The transfection experiment was repeated three times independently (1st, 2nd and 3rd). The average quantitative value of two holes was obtained, and the relative expression of HBsAg in E2 mutant plasmids (pHBV1.3B/C-E2N) was compared with genotype-matched pHBV1.3B/C by Fisher's LSD test.
